# Comparative analysis of oncogenic genes revealed unique evolutionary features of field Marek's disease virus prevalent in recent years in China

**DOI:** 10.1186/1743-422X-8-121

**Published:** 2011-03-15

**Authors:** Mingxing Tian, Yang Zhao, Yan Lin, Nianli Zou, Cheng Liu, Ping Liu, Sanjie Cao, Xintian Wen, Yong Huang

**Affiliations:** 1 College of Veterinary Medicine, Sichuan Agricultural University, Ya'an, Sichuan, 625014, PR China; 2 Key Laboratory of Animal Disease and Human Health of Sichuan Province, Sichuan Agricultural University, Ya'an, Sichuan, 625014, PR China

## Abstract

**Background:**

Marek's disease (MD) is an economically important viral disease of chickens caused by Marek's disease virus (MDV), an oncogenic herpesvirus. This disease was well controlled since the widespread use of commercial vaccines, but field MDVs have shown continuous increasing in virulence and acquired the ability to overcome the immune response induced by vaccines. Nowadays, MD continues to be a serious threat to poultry industry, isolation and characterization of MDVs are essential for monitoring changes of viruses and evaluating the effectiveness of existing vaccines.

**Results:**

Between 2008 and 2010, 18 field MDV strains were isolated from vaccinated chicken flocks in Sichuan province, China. Three oncogenic genes including Meq, pp38 and vIL-8 genes of the 18 isolates were amplified and sequenced. Homology analysis showed that the deduced amino acid sequences of these three genes exhibit 95.0-98.8%, 99.3-100% and 97.0-98.5% homology respectively with these of other reference strains published in GenBank. Alignment analysis of the nucleotide and deduced amino acid sequences showed that four amino acid mutations in Meq gene and two amino acid mutations in vIL-8 gene displayed perfect regularity in MDVs circulating in China, which could be considered as features of field MDVs prevalent in recent years in China. In addition, one amino acid mutation in pp38 gene can be considered as a feature of virulent MDVs from USA, and three amino acid mutations in Meq gene were identified and unique in very virulent plus (vv+) MDVs. Phylogenetic analysis based on Meq and vIL-8 protein sequences revealed that field MDVs in China evolved independently. Virulence studies showed that CVI988 could provide efficient protection against the field MDVs epidemic recently in China.

**Conclusions:**

This study and other published data in the GenBank have demonstrated the features of Meq, pp38 and vIL-8 genes of MDVs circulating in recent years in Sichuan, China. Mutations, deletions or insertions were observed in these three genes, and some mutations could be considered as the unique marks of the MDVs circulating presently in China. The paper supplies some valuable information concerning the evolution of MDV which is useful for the vaccine development and control of MD in China.

## Background

Marek's disease virus (MDV) is an oncogenic cell-associated α-herpesvirus that causes lymphoproliferative disease in the domestic chickens resulting in T-cell lymphomas in visceral organs and peripheral nerves as early as 3-4 weeks postinfection, the pathogenesis of MD has recently been reviewed in detail [1,2]. Early events involve lytic infection of B-cells followed by latent infection and oncogenic transformation of T-cells [3,4]. Transformed T-cells then infiltrate lymphoid tissues, peripheral nerves and visceral organs, usually leading to stunting and death of affected birds.

MDV strains (MDVs) have been classified into three serotypes that have major differences not only in the genome but also in the biological features. Serotype 1 MDVs includes all the oncogenic strains and their attenuated forms; serotype 2 are non-oncogenic viruses isolated in chickens; and serotype 3 are non-oncogenic virus isolated in turkeys, generally known as herpesvirus of turkeys or HVT [5]. Among those three serotypes, only serotype 1 are oncogenic, which can be further classified into four pathotypes including mild (m), virulent (v), very virulent (vv) to very virulent plus (vv+) strains [6-8]. MD was first described by Josef Marek in 1907 as a polyneuritis affecting mainly old chickens with low morbidity and negligible mortality, but acute MD became the predominant form in most countries that had a well-developed poultry industry in 1960 s. After the introduction and widespread use of HVT vaccines, the disease was then well controlled. During the 1970 s, there was a decrease in the efficiency of HVT vaccine due to interference from homologous maternal antibodies and the emergence of MDV field strains of increased virulence. In the mid-1980 s, a bivalent vaccine (a mixture of HVT and the serotype 2 strain SB-1) was introduced, and this provided better protection than either of the individual components used alone [9], a phenomenon known as protective synergism. With further increase in virulence of field viruses [7], the "Rispens" vaccine (attenuated serotype 1 strain CVI988) [10,11] was introduced for widespread use in the 1990 s. Recently, some reports have showed the failure of Rispens vaccine in Europe, when used either alone or in combination with MDV-2 and/or -3 vaccines, suggesting the emergence of hypervirulent strains [12,13].

To update, the molecular mechanism for MDV oncogenicity and pathogenicity remain largely unknown. Moreover, virus-encoded factors contributing to the enhanced virulence of MDV field strains remain uncharacterized. Several genes unique for MDVs have been identified [14-17], including latency associated transcripts (LATs), Meq (Marek's EcoQ), vIL-8, viral lipase, pp38/pp24, the 1.8 kb gene family, telomerase RNA (vTR) and MDV-encoded microRNAs [18]. Some of these genes are present only in serotype 1, and others may have homologues in MDV serotype 2 and/or HVT. Among those genes, Meq, pp38 and vIL-8 genes were reported to have the greatest possibility to be associated with viral oncogenicity and pathogenicity. Therefore, isolation of filed MDVs and sequence analysis of these genes can help us decipher the molecular characters of field MDVs epidemic recently in China.

In recent years, isolation and sequence analysis of MDVs in China were frequently reported in breeder or layer flocks which had been vaccinated by HVT or CVI988, all results showed that there are virulent MDVs circulated in China [19,20]. In several of these reports, the incidence rate of MD could reach up to 15%-60%. However, the nature of MDVs circulated in Sichuan was not clear. In this paper, 18 MDVs were isolated in Sichuan from clinical outbreaks that occurred in the period of 2008-2010, then the Meq, pp38 and vIL-8 genes of the isolates were sequenced and aligned, and phylogenetic analysis together with other published isolates was carried out. In addition, challenge test to 10-day-old SPF chickens was also performed to test the efficacy of MDV vaccines used commercially now. This study could provide useful information as to the nature of MDVs circulating in China, and the significance of this study in MDV prevention was also discussed.

## Methods

### Samples collection and MDV detection by polymerase chain reaction (PCR)

563 heparinized blood samples were collected from 23 chicken farms in Sichuan province of China, those farms were located in eight area of Sichuan, which are Chengdu, Luoshan, Mianyang, Deyang, Meishan, Ya'an, Xinjin and Ganzi. The DNA was extracted from blood by routine phenol-chloroform method and used as temples for PCR. The target gene of PCR was 132 bp repeated sequence of MDV-1 genome, which can distinguish field MDV strain from vaccine CVI988 strain. The sequences of the primers used for this purpose were R1: 5'-ATG CGA TGA AAG TGC TAT GGA G-3' and R2: 5'-ATC CCT ATG AGA AAG CGC TTG A-3' reported by Zhang et al [20]. Blood samples that contain two or three copies of 132 bp repeats by PCR were used for MDV isolation.

### Virus isolation

The lymphocytes were separated from the positive blood samples by lymphocyte separation medium (Tianjin Haoyang Biological Manufacture Co., Ltd), and were inoculated into primary duck embryo fibroblast (DEF) cells prepared from 11-day-old embryonated eggs, and incubated at 37°C with 5% CO_2 _for five days for each passage. After two blind passages, the existence of MDV in DEFs was verified by PCR detection of 132 bp repeated sequence. Only the positive samples having no contamination of Avian Leukosis virus (ALV) and Reticuloendotheliosis virus (REV) by PCR (Primers sequences were not shown) were used for further passages until the appearance of typical cytopathic effect between 48 and 144 h after inoculation. One representative MDV strain originated from a chicken farm was stored for further research.

### Primer for Meq, pp38 and vIL-8 genes

Three pairs of primers was designed on the basis of the DNA sequence of MDV-1 strain published in GenBank with the aid of computer software Primer Premier 5.0 (PREMIER Biosoft International, Palo Alto, CA, USA) (Table 1), primers were synthesized by Takara biotechnology (Dalian, China) Co., Ltd. The anticipated amplification segments for Meq, pp38 and vIL-8 were 1081 bp, 1006 bp and 887 bp long, respectively.

**Table 1:** Primers for amplification of oncogenic genes, amplicon sizes and DNA sequence accession numbers

Gene	5'-primer	3'-primer	Amplicon	Location	Strain	Reference
Meq	5'-GGCACGGTACAGGTGTAAAGAG-3'	5'-GCATAGACGATGTGCTGCTGAG-3'	1081 bp	133365-134445	GA	AF147806
pp38	5'-TCATCTTCAACCCACAGCCATCC-3'	5'-TCGCTTAATCTCCGCCTCCAAC-3'	1006 bp	127241-128246	CVI988	DQ530348
vIL-8	5'-GAGACCCAATAACAGGGAAATC-3'	5'-TAGACCGTATCCCTGCTCCATC-3'	887 bp	139304-140190	CVI988	DQ530348

### PCR amplification of oncogenic genes

Total DNA was extracted from DEFs using a sodium dodecyl sulfate (SDS) - proteinase K and phenol/chloroform protocol [21]. The concentration of DNA was adjusted to 1 μg/ml in water and stored at -20°C for further use. PCR amplification was carried out using 2 ul DNA as template in a total volume of 50 ul containing 25 ul 2× *Taq *PCR Mixture (TIANGEN, Beijing, China), 2 ul of 10 uM of each of the two primers, and 19 ul ddH_2_O. The optimum conditions for PCR of Meq and vIL-8 genes were as follows: 94°C for 4 min, 35 cycles at 94°C for 1 min, 56°C for 1 min, 72°C for 1.5 min, and final elongation at 72°C for 10 min. The optimum conditions for PCR of pp38 gene were as follows: 94°C for 4 min, 35 cycles at 94°C for 1 min, 60°C for 1 min, 72°C for 1.5 min, and final elongation at 72 °C for 10 min. The PCR product was analyzed in 0.9% agarose in Tris-borate-EDTA (TBE) buffer gel containing 0.5 mg/ml ethidium bromide.

### DNA cloning

Products of PCR reactions, corresponding to the predicted size of the target gene, were isolated from agarose gels and purified using E.Z.N.A.TM Gel Extraction Kit (Omega, USA). Purified PCR products were ligated with a TA cloning vector pMD19-T (TaKaRa, Japan) and transforming into DH5α Escherichia coli. (E. coli.) competent cell. Confirmation of clones containing recombinant plasmid was achieved by PCR and restriction enzyme (RE) digestion. The recombinant plasmid was sequenced by Sanggon Biotech (Shanghai, China) Co., Ltd.

### Sequence analysis of oncogenic genes

The obtained nucleotide sequences and the deduced amino acid sequences of oncogenic genes of MDV isolates were edited using the Editseq program in the Lasergene package (DNASTAR Inc, Madison, WI, USA), and compared with other reference MDVs for the homology analysis with the use of MegAlign program in the same package. Phylogenetic analysis of the amino acid sequences of Meq, pp38 and vIL-8 genes were performed with the neighbor-joining method using MEGA version 4.0. The bootstrap values were determined from 1000 replicates of the original data. 18 other MDV reference strains were chosen for comparison, including 0093, 0095, 0297, 0304, G2, GX070060, GX070060, GXY2, YLO40920, 3004, 814, CVI988, CU-2, GA, Md5, RB1B, 648A, and 584A. Of these, three strains were vaccine strains, nine strains were isolated from China and six strains were isolated from USA. Among these 18 reference strains, eighteen strains were used for comparison of Meq genes, six strains were used for comparison of pp38 genes and seven strains were used for comparison of vIL-8 genes. The MDV reference strains were retrieved from the GenBank database, and the backgrounds of the reference strains used in this study are listed in Table 2.

**Table 2:** MDV reference strains published in GenBank

MDV stains	Virulence	Geographic origin	Year of isolation	Accession number
0093	High virulence	Guangxi, China	2002	AF493550(M)
0095	High virulence	China	2002	AF493552(M)
0297	High virulence	China	2002	AF493553(M)
0304	High virulence	Guangxi, China	2002	AF493554(M)
G2	High virulence	Guangxi, China	2002	AF493556(M)
GX070060	High virulence	China	2008	EU427303(M)
GX070079	High virulence	China	2008	EU427304(M)
GXY2	High virulence	China	2007	EF546430(M)
YLO40920	High virulence	China	2005	DQ174459(M)
3004	Vaccine Strain	Russia	N/A	EU032468(M)
814	Vaccine strain	China	N/A	GU354326(M)
CVI988	Commercial Vaccine	Netherland	1972	DQ534538(M, V), DQ530348(P)
CU-2	Mild virulence	USA	N/A	EU499381(M, V, P)
GA	virulence	USA	1964	AF147806(M, P), AF065430(V)
Md5	Very virulence	USA	1979	AF243438(M, V, P)
RB1B	Very virulence	USA	1982	EF523390(M, V, P)
648A	Very virulence plus	USA	1997	AY362725(M), DQ534534(V)
584A	Very virulence plus	USA	Before 2000	DQ534532(M, V)

"M" refers to accession number of Meq gene; "P" refers to accession number of pp38 gene; "N" refers to accession number of vIL-8 gene; N/A, data not available.

### Virulence studies

In order to study the pathogenecity of MDVs isolated in Sichuan, one representative MDV isolates, LS strain, was used as challenge strain. 90 SPF chickens were randomly divided into three groups, 30 birds in HVT immunized group were vaccinated with 2000 PFU HVTs of FC126 strain (Harbin Weike Biotechnology Developmengt Co., Ltd.), 30 birds in CVI988 immunized group were vaccinated with 4000 PFU MDVs of CVI988/Rispens strain (Beijing Lingyu Biotechnology Co., Ltd.) and 30 birds in control group were inoculated with phosphate buffer (PBS) at the age of one day. All the birds in the same group were held solely in biosafety level 2 (BSL2) isolators in separate rooms with adlibitum access to feed and water and maintained under uniform standard management conditions, and inoculated intraperitoneally (i.p.) with 0.5 ml diluent of DEFs containing 4000 PFU LS MDVs at the age of ten days. Clinical signs and gross postmortem lesions as well as mortalities were recorded for a period of 60 days after virus challenge. SPSS 17.0 software was used to evaluate the data and determine the significant difference of the rate of tumor occurrences and mortality between the immune group and control group. Besides, organs of dead chickens in control group, such as liver, spleen, kidney, were sampled for histopathological diagnosis.

## Results

### PCR result of 132-bp repeats of blood samples

563 blood samples of 23 chicken farms were detected by PCR of 132 bp-repeat sequence of MDV-1 genome, and 145 blood samples were positive by PCR containing two or three copies of 132 bp-repeats, the positive detection rate reached 25.8%.

### Virus isolation

18 representative MDV typical strains were isolated from 18 of 23 chicken farms by using DEFs culturing, PCR detection showed that the 18 MDVs were free of ALV and REV. The isolated MDVs adapted to DEFs very well and could cause typical cytopathic effect (CPE) after 4-6 blind passages. The MDVs isolated from other five chicken farms were discarded because of the contamination of ALV or REV. The case histories of local strains are listed in Table 3.

**Table 3:** MDVs isolated since 2008 from flocks in different areas of Sichuan province, China

Strains	Source of isolation	Year of isolation	Chicken type	Age(days)	location	Accession number
LS	DEF	2008	Breeder	125	Leshan	HQ638149(M), HQ638163(P), HQ638183(V)
BY	DEF	2008	Tibetan breeder	121	Ganzi	HM991861(M), HQ638159(P), HQ638179(V)
YY	DEF	2008	Breeder	90	Ya'an	HQ638157(M), HQ638177(P), HQ638197(V)
YA	DEF	2008	Broiler	106	Ya'an	HQ638156(M), HQ638176(P), HQ638196(V)
MS01	DEF	2009	Breeder	135	Meishan	HQ638143(M), HQ638164(P), HQ638184(V)
MS53	DEF	2009	Breeder	234	Meishan	HQ638144(M), HQ638165(P), HQ638184(V)
MS54	DEF	2009	Breeder	234	Meishan	HQ638145(M), HQ638166(P), HQ638186(V)
MS67	DEF	2009	Breeder	245	Meishan	HQ638146(M), HQ638167(P), HQ638187(V)
NC01	DEF	2008	Breeder	220	Ya'an	HQ638147(M), HQ638168(P), HQ638188(V)
TQ20	DEF	2009	Broiler	66	Tianquan, Ya'an	HQ638151(M), HQ638171(P), HQ638191(V)
DY01	DEF	2009	Breeder	N/A	Deyang	HQ638141(M), HQ638160(P), HQ638180(V)
DY04	DEF	2009	Breeder	N/A	Deyang	HQ638142(M), HQ638161(P), HQ638181(V)
XJ01	DEF	2010	Breeder	110	Xinjin	HQ638154(M), HQ638174(P), HQ638194(V)
XJ03	DEF	2010	Breeder	110	Xinjin	HQ638155(M), HQ638175(P), HQ638195(V)
WS03	DEF	2010	Breeder	N/A	Deyang	HQ638152(M), HQ638172(P), HQ638192(V)
WS04	DEF	2010	Breeder	N/A	Deyang	HQ638153(M), HQ638173(P), HQ638193(V)
5079	DEF	2010	Breeder	130	Ya'an	HQ638140(M), HQ638158(P), HQ638178(V)
NC02	DEF	2010	Breeder	120	Ya'an	HQ638148(M), HQ638169(P), HQ638189(V)

"M" refers to accession number of Meq gene; "P" refers to accession number of pp38 gene; "N" refers to accession number of vIL-8 gene; N/A, data not available.

### Copy numbers of 132-bp repeats in 18 field MDVs

**Figure 1:**
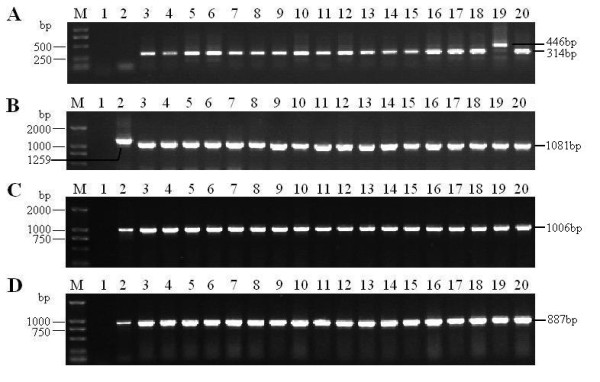
**PCR products of 132 bp repeats, Meq, pp38 and vIL-8 gene of 18 MDV isolates from China**. Lane M: DL2000 Marker; Lane 1-20: ddH_2_0, CVI988, LS, BY, YY, MS01, MS53, MS54, MS67, NC01, NC02, TQ20, DY01, DY04, XJ01, XJ4, WS03, WS04, YA, and 5079, respectively; A. analysis of copy numbers of 132 bp repeats in 18 MDV isolates; B. Amplification of Meq gene of 18 MDV isolates; C. Amplification of pp38 gene of 18 MDV isolates; D. Amplification of vIL-8 gene of 18 MDV isolates.

The copy numbers of 132 bp repeats in the genome of field isolates were examined, the length of one copy of 132-bp repeats is 182 bp long, containing one 132-bp repeat and 50 nucleotides from primers, the length of two copies of 132 bp repeats are 314 bp, and the length of three copies of 132 bp repeats are 446 bp by analogy. In 18 field MDVs, the YA strain is the only one containing three copies of 132 bp repeats, the other isolates containing two copies (Figure 1-A).

### Results of PCR amplification of Meq, pp38 and vIL-8 genes

The total DNA of DEFs infected by field MDVs was extracted to be used as PCR template, and the complete sequence of Meq, pp38 and vIL-8 genes of 18 field MDVs was successfully amplified (Figure 1-B, C and 1D).

### High nucleotides and amino acids identities of Meq, pp38 and vIL-8 genes between isolated MDVs and other reference MDVs

Meq, pp38 and vIL-8 gene sequences of the 18 MDV isolates were delineated and submitted to the GenBank database (Table 3).

Homology analysis of Meq gene showed that the homology of the nucleotide and deduced amino acid sequences of the 18 Sichuan isolates were 99.7-100.0% and 99.1-100.0%, respectively, and were 98.2-100% and 97.1-100% between MDVs from Sichuan and these from other areas of China, but were 97.7-99.6% and 95.0-98.8% between MDVs from China and those from other countries.

For pp38 gene, the homology of the nucleotide and deduced amino acid sequences of the 18 Sichuan isolates were 99.7-100.0% and 99.3-100.0%, respectively, and were 99.4-100% and 99.3-100% between MDVs from Sichuan and those from other countries. However, no sequences of pp38 gene of MDVs isolated in other areas of China are available in GenBank now.

As for the vIL-8 gene, the homology of the nucleotide and deduced amino acid sequences of the 18 isolates were 99.5-100.0% and 98.5-100.0%, respectively, and were 99.0-99.5% and 97.0-98.5% between MDVs from Sichuan and those from other countries. However, no sequences of vIL-18 gene of MDV isolated in other areas of China are available in GenBank now.

### Alignment analysis of nucleotide and deduced amino acid sequences of Meq, pp38 and vIL-8 genes

**Figure 2:**
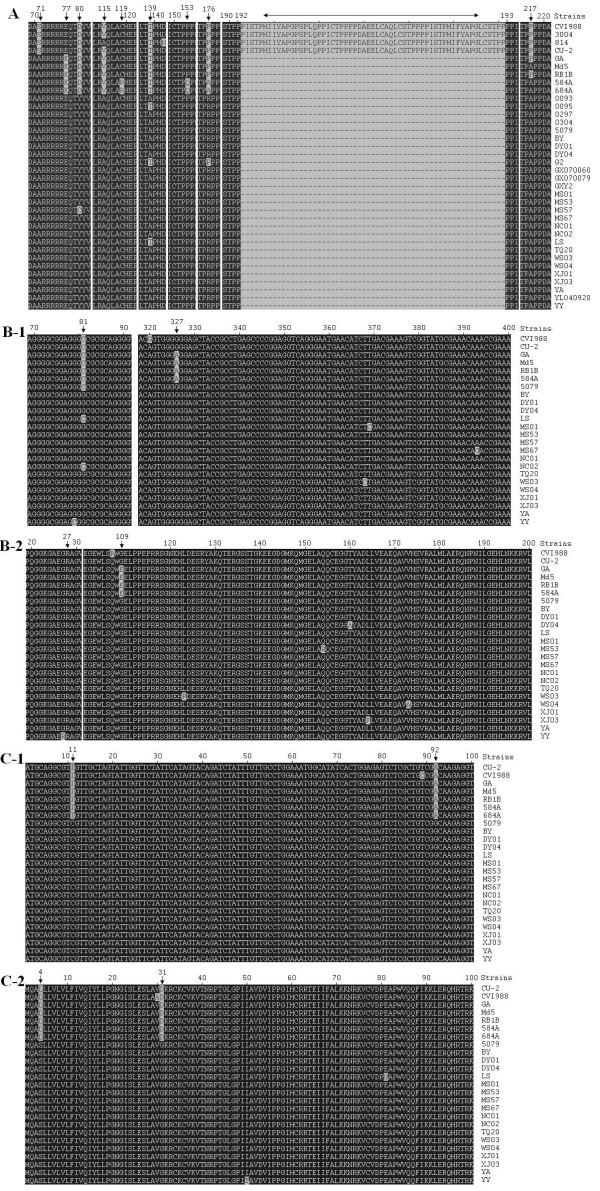
**Comparison of deduced amino acid sequences of three oncogenic genes**. A. Alignment analysis of amino acid sequences of Meq gene; B-1. Alignment analysis of nucleotide sequences of pp38 gene; B-2. Alignment analysis of amino acid sequences of pp38 gene; C-1. Alignment analysis of nucleotide sequences of of vIL-8 gene; C-2. Alignment analysis of amino acid sequences of vIL-8 gene. Vertical arrow refers to mutation of nucleotide or amino acid (aa) and two-way arrow refers to deletion of 59aa.

Alignment analysis of Meq complete nucleotide and deduced amino acid sequences of the 18 isolates and 18 published MDVs were performed. Nucleotide mutations, insertions, and/or deletions were observed when GA strain was used as reference strain. The amino acid mutation in the Meq gene of MDVs displayed regularity at nine positions, including 71, 77, 80, 115, 119, 139, 153, 176 and 217, and amino acid mutations at position 80, 115, 139 and 176 occurred in most field MDVs from China. The amino acid mutations at positions 119, 153 and 176 were unique in very virulence plus (vv+) strains from USA 684A and 584A. Besides, a 59aa insertion was also observed in the Meq gene of vaccine or mild virulent strains between position 192 and 193 of virulent strains (numbering using amino acid sequence of GA as reference) (Figure 2A).

As for pp38 gene, the complete nucleotide sequences and deduced amino acid sequences of 18 isolates were compared with these of other 6 reference MDVs, and the results showed that the mutation displayed regularity at position 81(C→G) and 327(A→G) (Figure 2B-1), resulting in a nonsense amino acid mutation at position 27 (glycine), and a sense amino acid mutation at position 109 (glutamate → glycine) of virulent MDVs from USA (Figure 2B-2).

As for vIL-8 gene, the complete nucleotide sequences and deduced amino acid sequences of 18 isolates were compared with these of other 7 reference MDVs, and the results showed that mutation at position 11(T → C) and 92 (A → G) (Figure 2C-1) were common in 18 isolates, resulting in two amino acid mutations at position 4 (leucine → serine) and 31 (aspartate → glycine) of MDVs from China (Figure 2C-2).

### Phylogenetic analysis based on deduced amino acid sequences of Meq, pp38 and vIL-8 genes

**Figure 3:**
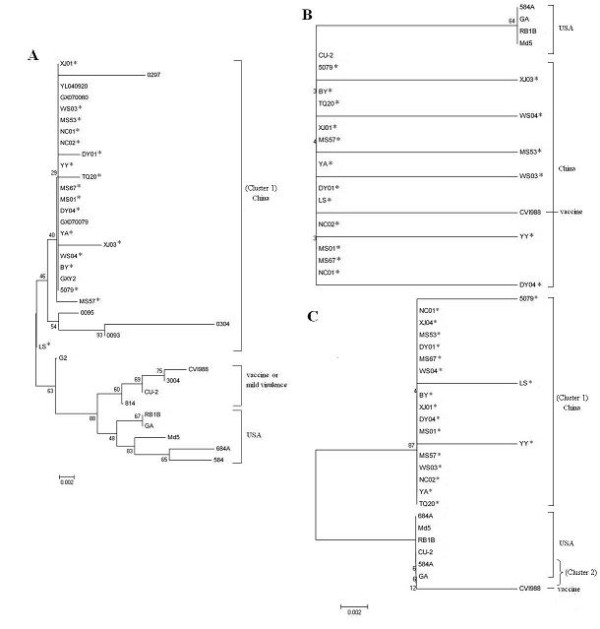
**Phylogenetic analysis on Meq, pp38 and vIL-8 gene sequences of 18 Sichuan isolates and other reference MDVs**. The phylogenetic tree was constructed using the MEGA version 4.0 by the neighbor-joining method with 1000 bootstrap replicates. Asterisks indicate the 18 isolates from Sichuan.

Phylogenetic analysis on the Meq and vIL-8 sequences of 18 isolates and other 18 reference strains showed that the analyzed 36 MDVs could be separated two groups (cluster 1 and cluster 2), 18 MDVs isolated from Sichuan and other virulent MDVs from China were included in cluster 1; while, vaccine strains, mild virulent strains and virulent strains from USA were included in cluster 2 (Figure 3A, C). In addition, for the phylogenetic tree of Meq gene, the MDVs in cluster 2 could be further grouped into 2 branches, virulent strain from USA were included in one branch; while, vaccine strains or mild virulent strains were included in another branch (Figure 3A). No obvious cluster or branch was observed in the phylogenetic tree of pp38 gene (Figure 3B).

### Virulence studies

**Figure 4:**
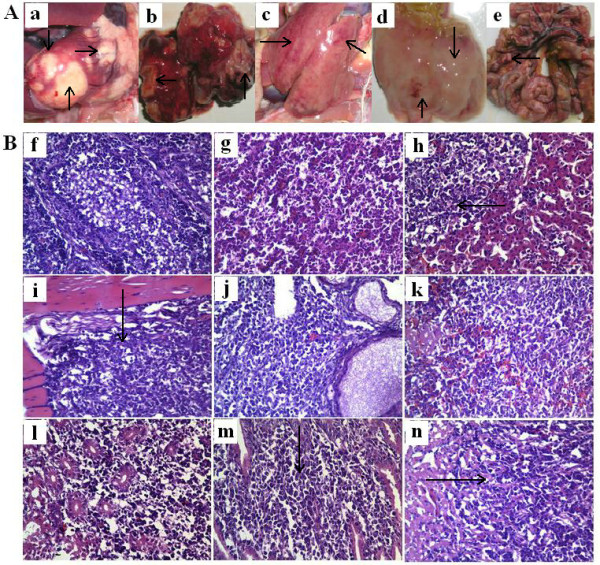
**Clinical and histopathologic observation of diseased chickens in control group infected by MDV LS strain**. A. Clinical anatomical change, "a" to "e" were heart, lung, liver, stomachus glandularis, intestinal tract, respectively, and tumors were observed in these tissues, even in the intestinal tract and mesenterium; B. Diagnosis by histopathologic slices (HE 400×). All tissues were fixed in 10% formalin paraffin and embedded, and 5 μm sections were stained with haematoxylin and eosin (HE), for light microscopy, "f" to "n" refer to bursa of fabricius, lung, liver, skeletal muscle, ovary, spleen, kidney, glandular stomach mucosa, cardiac muscle. Arrows show that multimorphology lymphocytes infiltrated patically and formed tumors (h, i, m, n), and other organs were infiltrated by lymphocytes extensively and their organizations were completely destroyed (f, g, j, k, i).

No clinical sign was observed during the first two week post challenge. Chickens become depressed and comatose since two weeks post challenge in HVT immune group and control group, and some infected chickens may also exhibit flaccid neck paralysis; while no obvious clinical sign was observed in CVI988 immunized group. The earliest death appeared on 18 days post challenge in control group, and 21 days post challenge in HVT immunized group. The peak of death came on 3 weeks post challenge in control group, but 4 weeks post challenge in HVT immunized group. Lymphomas occurred in a variety of organs and tissues after postmortem examination, especially in the lung and heart (Figure 4-A). The rate of tumor occurrences and mortality were 80.00% and 76.67% in control group, while 76.67% and 70.00% in HVT immunized group. The difference of the rate of tumor occurrences and the mortality between the HVT immunized group and control group were not significant (P > 0.05). In addition, there was no death or tumor occurrence in chicken of CVI988 immunized group during the experiment period.

### Histopathological observation of infected SPF chicken

Histopathologic slices showed that multimorphous and immature lymphocytes infiltrated and proliferated in almost all kinds of organs, including cardiac muscle, hepatic lobule, skeletal muscle, lung, spleen, ovary, kidney, bursa of fabricius and glandular stomach mucosa. Most histiocytes of the digestive system showed swell, degeneration, necrosis and almost all the structure was completely destroyed (Figure 4-B).

## Discussion

Marek's disease (MD) is currently one of the important contagious diseases in poultry industry. It not only causes the death of birds directly, but also causes immunosuppression of infected chickens which are more sensitive to other pathogens such as E. coli. Although CVI988 vaccine [10] is widely used now in China, but immune failure still occurred in some flocks. It had been reported that the virulence of MDV isolates have increased in the last four decades [7], and some of the recent isolates are more pathogenic for chickens than MDVs isolated before [22]. The emergence of MDVs of increasing virulence is a significant problem for the poultry industry.

MDV is a member of the genus Mardivirus that consists of serotypes 1 and 2 (MDV-1 and MDV-2) as well as serotype 3 or herpesvirus of turkeys (HVT) [23]. Among them, the only serotype 1 is oncogenic, and some of the unique genes such as Meq (MDV EcoRI-Q) [24], pp38 [25], vIL-8 (viral interleukin-8) [26] were reported to be associated with viral oncogenicity and pathogencity.

In this study, homology comparison of the nucleotide and deduced amino acid sequences of Meq, pp38 and vIL-8 of 18 isolates and other reference strains were conducted, and high nucleotides and amino acids identities of these three genes between isolated MDVs and other reference MDVs were observed, indicating that the genetic stability of these three genes was high in recent years. Currently, Meq gene is most likely considered to be the factor to induce tumors. It encodes 339 amino acids and contains a basic leucine zipper (bZIP) domain at the N terminal closely resembling the *jun/fos *oncogene family [24], and its transactivation can lead to cell transformation [27,28]. The length of Meq gene of JM, CU-2 and CVI988 was 177bp longer than these of virulent MDVs [29,30], which result in a 59 aa insertion. It is reported to have a suppressive effect on the Meq expression [31]. After sequence alignment, this insertion was also found in Chinese vaccine 814 and Russian vaccine strains 3004, but not found in all 18 field isolates. In addition, it had been reported previously that amino acid change at position 71 (serine → alanine) and 77 (glutamate → lysine) was the feature of high virulent MDVs [32]. In this study, the same mutation at position 71 was also observed in all wild strains from China, but the mutation at position 77 was not observed in field isolates from China, and this mutation seemed to be a feature of virulent MDVs from USA. Besides, it was also found that some amino acid mutations at position 80, 115, 139 and 176 in most isolates from China displayed perfect regularity. Mutation at position 80 (aspartate → tyrosine), 115 (valine → alanine), 139 (threonine → alanine), and 176 (proline → arginine) accounted for 96.3% (26/27), 100% (27/27), 90.0% (24/27) and 96.3% (26/27) of all field MDVs (not including vaccine 814 strain) from China, respectively. The mutation in position 80, 115, 139, 176 of Meq protein could be used as virulent genetic characteristics of the circulating MDVs MDV in China. Moreover, it was observed that the three amino acids at position 119 (arginine), 153 (glutamine) and 176 (alanine) were unique in very virulence plus (vv+) strains 684A and 584A, but we can not determine that whether this is the character of all vv+ MDVs.

pp38 codes a 38Ku phosphoprotein and plays a role in viral reactivation in latent period [33,34] and cell transformation [35,36]. Minor differences in amino acid sequences of different MDVs have been observed for pp38. Originally, it was believed that pp38 was not expressed in CVI988 [37]; however, it was showed to be present in CVl988 later, and glutamine in position 107 was replaced by arginine as defined by monoclonal antibody (MAb) H19 [38,39]. In this study, after alignment of the amino acid sequence of pp38 gene of 25 MDVs, it was found that no mutation at position 107 was observed in all the field isolates, but glutamate at position 109 in virulent MDVs (GA, RB1B, 584A and 648A) from USA were replaced by Glycine in field isolates from China. So the mutation (Glutamate → glycine) in position 109 can be considered as a feature of virulent MDVs isolated from USA.

The vIL-8 gene locates in the long repeat region and was originally identified as a spliced Meq variant [40]. This gene consists of 3 exons and is expressed during cytolytic infection. vIL-8 attracts T cells, especially after vIL-8 receptors are up-regulated by interferon-γ (lFN-γ). vIL-8 may be important for the switch of infection from B to T lymphocytes[41]. This gene was very conservative in 648A, RB1B, MD11 and GA strains [42], but after comparison of the amino acid sequence, two special point mutations at position 4 (leucine→ serine) and 31 (aspartate → glycine) were found only in the field MDVs from China. We speculate that the point mutations at position 4 and 31 could be considered as the features of field MDVs in China. 

Phylogenetic tree, based on the Meq and vIL-8 amino acid sequences, revealed that the field MDVs from China formed an independent cluster, while vaccine strains, mild virulent MDVs and virulent MDVs from USA formed another cluster. In addition, in the phylogenetic tree of Meq, the MDVs in the latter cluster could be further divided into two different branches. This result implied that field MDVs from China may evolve independently, and genetic difference of MDVs displayed in phylogenetic tree of the Meq was more obvious than these of vIL-8 and pp38 gene. Meq gene could be a gene of priority for Phylogenetic analysis.

Virulence study in this study revealed that virulent MDVs prevalent in China in recent years could break through the protection provided by HVT vaccine. Histopathologic diagnosis of the diseased chicken showed multimorphous and immature lymphocytes infiltrated and proliferated in all kinds of organs, leading to complete destruction of the structure of organs and tissues. CVI988/Rispens vaccine is a better choice for immunization. Currently, MD occasionally occurred in some chicken farms immunized with CVI988 vaccines in China; however, result of virus isolation and virulence test did not show that the very virulence plus (vv+) strain, such as 684A in USA, was epidemic in China, and the immune failure may be due to the improper selection of vaccines and the incorrect way of vaccination.

In conclusion, high nucleotides and amino acids identities of Meq, pp38 and vIL-8 genes between 18 MDV isolates and other reference MDVs was observed. Four mutations at position 80, 115, 139 and 176 of the amino acid sequence of Meq gene, two mutations at position 4 and 31 of the amino acid sequence of vIL-8 gene can be considered as main features of field MDVs prevalent in recent years in China; one mutation at position 109 in amino acid sequence of pp38 gene can be considered as a feature of virulent MDVs isolated from USA. Phylogenetic analysis of Meq genes could provide the evolution difference of different MDVs. CVI988/Rispens vaccine could provide enough protection against the challenge of prevalent MDVs, and should be widely used commercially. Nevertheless, as new variant strains may emerge in the future, constant surveillance of new filed MDVs is necessary to reveal the character of epidemic MDVs and to develop better vaccines and control program of MD.

## Competing interests

The authors declare that they have no competing interests.

## Authors' contributions

MXT: Study design, performed the experiments, interpretation of the data and wrote the manuscript. YZ, YL, NL, CL, PL, SC, XW and YH: helped in experiments and drafted the manuscript. All authors read and approved the final manuscript.

## References

[B1] Calnek BW Pathogenesis of Marek's disease virus infection Curr Top Microbiol Immunol 2001 255 25 55 11217426 10.1007/978-3-642-56863-3_2

[B2] Baaten BJ Butter C Davison TF Study of host-pathogen interactions to identify sustainable vaccine strategies to Marek's disease Vet Immunol Immunopathol 2004 100 165 177 10.1016/j.vetimm.2004.04.009 15207454

[B3] Calnek BW Schat KA Ross LJ Chen CL Further characterization of Marek's disease virus-infected lymphocytes. II. In vitro infection Int J Cancer 1984 33 399 406 10.1002/ijc.2910330319 6321365

[B4] Shek WR Calnek BW Schat KA Chen CH Characterization of Marek's disease virus-infected lymphocytes: discrimination between cytolytically and latently infected cells J Natl Cancer Inst 1983 70 485 491 6300499

[B5] Bulow VV Biggs PM Differentiation between strains of Marek's disease virus and turkey herpesvirus by immunofluorescence assays Avian Pathol 1975 4 133 146 18777301 10.1080/03079457509353859

[B6] Witter RL Characteristics of Marek's disease viruses isolated from vaccinated commercial chicken flocks: association of viral pathotype with lymphoma frequency Avian Dis 1983 27 113 132 10.2307/1590377 6303287

[B7] Witter RL Increased virulence of Marek's disease virus field isolates Avian Dis 1997 41 149 163 10.2307/1592455 9087332

[B8] Witter RL Calnek BW Buscaglia C Gimeno IM Schat KA Classification of Marek's disease viruses according to pathotype: philosophy and methodology Avian Pathol 2005 34 75 90 10.1080/03079450500059255 16191686

[B9] Witter RL Lee LF Polyvalent Marek's disease vaccines: safety, efficacy and protective synergism in chickens with maternal antibodies Avian Pathol 1984 13 75 92 10.1080/03079458408418510 18766823

[B10] Rispens BH van Vloten H Mastenbroek N Maas HJ Schat KA Control of Marek's disease in the Netherlands. I. Isolation of an avirulent Marek's disease virus (strain CVI 988) and its use in laboratory vaccination trials Avian Dis 1972 16 108 125 10.2307/1588905 4337307

[B11] de Boer GF Groenendal JE Boerrigter HM Kok GL Pol JM Protective efficacy of Marek's disease virus (MDV) CVI-988 CEF65 clone C against challenge infection with three very virulent MDV strains Avian Dis 1986 30 276 283 10.2307/1590529 3015113

[B12] Burgess SC Young JR Baaten BJ Hunt L Ross LN Parcells MS Kumar PM Tregaskes CA Lee LF Davison TF Marek's disease is a natural model for lymphomas overexpressing Hodgkin's disease antigen (CD30) Proc Natl Acad Sci USA 2004 101 13879 13884 10.1073/pnas.0305789101 15356338 PMC518847

[B13] Schumacher D Tischer BK Teifke JP Wink K Osterrieder N Generation of a permanent cell line that supports efficient growth of Marek's disease virus (MDV) by constitutive expression of MDV glycoprotein E J Gen Virol 2002 83 1987 1992 12124462 10.1099/0022-1317-83-8-1987

[B14] Afonso CL Tulman ER Lu Z Zsak L Rock DL Kutish GF The genome of turkey herpesvirus J Virol 2001 75 971 978 10.1128/JVI.75.2.971-978.2001 11134310 PMC113993

[B15] Izumiya Y Jang H Ono M Mikami T A complete genomic DNA sequence of Marek's disease virus type 2, strain HPRS24 Current topics in microbiology and immunology 2001 255 191 221 11217423 10.1007/978-3-642-56863-3_8

[B16] Lupiani B Lee L Reddy S Protein-coding content of the sequence of Marek's disease virus serotype 1 Current topics in microbiology and immunology 2001 255 159 190 11217422 10.1007/978-3-642-56863-3_7

[B17] Lee L Wu P Sui D Ren D Kamil J Kung H Witter R The complete unique long sequence and the overall genomic organization of the GA strain of Marek's disease virus Proceedings of the National Academy of Sciences of the United States of America 2000 97 6091 10.1073/pnas.97.11.6091 10823954 PMC18563

[B18] Saif YM Barnes HJ Diseases of poultry 2008 12 Ames, Iowa: Blackwell Pub. Professional 452 514

[B19] Zuo T Zhao Z Wei P Wei X Li Y Mo M Isolation and identification of a field isolate of Marek's disease virus with acute oncogenicity Chinese Journal of Virology 2007 23 218 223

[B20] Chen M Payne WS Hunt H Zhang H Holmen SL Dodgson JB Inhibition of Marek's disease virus replication by retroviral vector-based RNA interference Virology 2008 377 265 272 10.1016/j.virol.2008.03.019 18570965

[B21] Sambrook J Russell DW Molecular cloning: a laboratory manual 2001 3 Cold Spring Harbor, N.Y.: Cold Spring Harbor Laboratory Press

[B22] Witter RL Protective efficacy of Marek's disease vaccines Curr Top Microbiol Immunol 2001 255 57 90 11217428 10.1007/978-3-642-56863-3_3

[B23] Davison AJ Evolution of the herpesviruses Vet Microbiol 2002 86 69 88 10.1016/S0378-1135(01)00492-8 11888691

[B24] Jones D Lee L Liu JL Kung HJ Tillotson JK Marek disease virus encodes a basic-leucine zipper gene resembling the fos/jun oncogenes that is highly expressed in lymphoblastoid tumors Proc Natl Acad Sci USA 1992 89 4042 4046 10.1073/pnas.89.9.4042 1315048 PMC525628

[B25] Cui ZZ Lee LF Liu JL Kung HJ Structural analysis and transcriptional mapping of the Marek's disease virus gene encoding pp38, an antigen associated with transformed cells J Virol 1991 65 6509 6515 1658357 10.1128/jvi.65.12.6509-6515.1991 PMC250698

[B26] Parcells MS Lin SF Dienglewicz RL Majerciak V Robinson DR Chen HC Wu Z Dubyak GR Brunovskis P Hunt HD Marek's disease virus (MDV) encodes an interleukin-8 homolog (vIL-8): characterization of the vIL-8 protein and a vIL-8 deletion mutant MDV J Virol 2001 75 5159 5173 10.1128/JVI.75.11.5159-5173.2001 11333897 PMC114921

[B27] Levy AM Izumiya Y Brunovskis P Xia L Parcells MS Reddy SM Lee L Chen HW Kung HJ Characterization of the chromosomal binding sites and dimerization partners of the viral oncoprotein Meq in Marek's disease virus-transformed T cells J Virol 2003 77 12841 12851 10.1128/JVI.77.23.12841-12851.2003 14610205 PMC262596

[B28] Ajithdoss DK Reddy SM Suchodolski PF Lee LF Kung HJ Lupiani B In vitro characterization of the Meq proteins of Marek's disease virus vaccine strain CVI988 Virus Res 2009 142 57 67 10.1016/j.virusres.2009.01.008 19189855

[B29] Chang KS Ohashi K Onuma M Diversity (polymorphism) of the Meq gene in the attenuated Marek's disease virus (MDV) serotype 1 and MDV-transformed cell lines J Vet Med Sci 2002 64 1097 1101 10.1292/jvms.64.1097 12520100

[B30] Lee LF Wu P Sui D Ren D Kamil J Kung HJ Witter RL The complete unique long sequence and the overall genomic organization of the GA strain of Marek's disease virus Proc Natl Acad Sci USA 2000 97 6091 6096 10.1073/pnas.97.11.6091 10823954 PMC18563

[B31] Chang KS Ohashi K Onuma M Suppression of transcription activity of the MEQ protein of oncogenic Marek's disease virus serotype 1 (MDV1) by L-MEQ of non-oncogenic MDV1 J Vet Med Sci 2002 64 1091 1095 10.1292/jvms.64.1091 12520099

[B32] Shamblin CE Greene N Arumugaswami V Dienglewicz RL Parcells MS Comparative analysis of Marek's disease virus (MDV) glycoprotein-, lytic antigen pp38- and transformation antigen Meq-encoding genes: association of Meq mutations with MDVs of high virulence Vet Microbiol 2004 102 147 167 10.1016/j.vetmic.2004.06.007 15327791

[B33] Pratt WD Cantello J Morgan RW Schat KA Enhanced expression of the Marek's disease virus-specific phosphoproteins after stable transfection of MSB-1 cells with the Marek's disease virus homologue of ICP4 Virology 1994 201 132 136 10.1006/viro.1994.1273 8178477

[B34] Yamaguchi T Kaplan SL Wakenell P Schat KA Transactivation of latent Marek's disease herpesvirus genes in QT35, a quail fibroblast cell line, by herpesvirus of turkeys J Virol 2000 74 10176 10186 10.1128/JVI.74.21.10176-10186.2000 11024146 PMC102056

[B35] Ross NL T-cell transformation by Marek's disease virus Trends Microbiol 1999 7 22 29 10.1016/S0966-842X(98)01427-9 10068994

[B36] Gimeno IM Witter RL Hunt HD Reddy SM Lee LF Silva RF The pp38 gene of Marek's disease virus (MDV) is necessary for cytolytic infection of B cells and maintenance of the transformed state but not for cytolytic infection of the feather follicle epithelium and horizontal spread of MDV J Virol 2005 79 4545 4549 10.1128/JVI.79.7.4545-4549.2005 15767457 PMC1061578

[B37] Witter R Silva R Lee L New serotype 2 and attenuated serotype 1 Marek's disease vaccine viruses: selected biological and molecular characteristics Avian Diseases 1987 31 829 840 10.2307/1591039 2831870

[B38] Davidson I Borovskaya A Perl S Malkinson M Use of the polymerase chain reaction for the diagnosis of natural infection of chickens and turkeys with Marek's disease virus and reticuloendotheliosis virus Avian Pathology 1995 24 69 94 10.1080/03079459508419050 18645767

[B39] Cui Z Qin A Lee L Wu P Kung H Construction and characterization of a H19 epitope point mutant of MDV CVI988/Rispens strain Acta virologica 43 169 10696440

[B40] Peng Q Shirazi Y Characterization of the protein product encoded by a splicing variant of the Marek's disease virus Eco-Q gene (Meq) Virology 1996 226 77 82 10.1006/viro.1996.0629 8941324

[B41] Schat KA Xing Z Specific and nonspecific immune responses to Marek's disease virus Dev Comp Immunol 2000 24 201 221 10.1016/S0145-305X(99)00073-7 10717288

[B42] Zhang Y Qin A Expression of viral interleukin 8 gene of Marek's disease virus and its biological characterization 2003 Yanzhou University, Veterinay College

